# Clinical features of acute attacks, chronic symptoms, and long-term complications among patients with acute hepatic porphyria in Japan: a real-world claims database study

**DOI:** 10.1186/s13023-023-02913-0

**Published:** 2023-12-08

**Authors:** Yutaka Horie, Yuka Yasuoka, Tomohide Adachi

**Affiliations:** 1 Department of Gastroenterology, Saiseikai Gotsu General Hospital, Shimane, Japan; 2 Alnylam Japan KK, Pacific Century Place Marunouchi 11th Floor 1-11-1 Marunouchi, Chiyoda-ku, Tokyo, 100-6211 Japan; 3 Department of General Medicine and Neurology, Saiseikai Central Hospital, Tokyo, Japan

**Keywords:** Porphyria, Attacks, Chronic symptoms, Complications, Japan, Hospitalizations, Hemin, Incidence, MDV, Acute

## Abstract

**Background:**

Acute hepatic porphyria (AHP) is a family of rare genetic diseases, including acute intermittent porphyria, variegate porphyria, hereditary coproporphyria, and delta-aminolevulinic acid dehydratase-deficient porphyria. The objective of this retrospective cohort study was to provide information on the clinical features of AHP in Japan—including acute attacks, chronic symptoms, and long-term complications.

**Methods:**

Patients with AHP between April 2008 and June 2020 were selected from Japan’s Medical Data Vision claims database. Patients with AHP were matched 1:10, by sex and age, to patients without AHP. The outcomes were evaluated overall, for patients age ≥ 55 years, and for the matched population.

**Results:**

A total of 391 patients with AHP were included from the Japanese Medical Data Vision database. During the observation period (April 2008–June 2020), 18.2% (71/391) of patients experienced 1 acute attack and 10.5% (41/391) experienced ≥ 2 attacks. Chronic symptoms with rates ~ 10% or higher in the AHP population compared with the matched population included neurotic, stress-related, and somatoform disorders (21.7% vs. 6.7% [15.0% difference]); sleep disorders (23.0% vs. 9.9% [13.1% difference]); other and unspecified abdominal pain (13.6% vs. 3.7% [9.9% difference]); and nausea and vomiting, excluding chemotherapy-induced emesis (17.9% vs. 8.1% [9.8% difference]). Long-term complications with higher incidence rates in the AHP population compared with the matched population included fibrosis and cirrhosis of liver (15.9% vs. 3.0% [12.9% difference]), polyneuropathies and other disorders of the peripheral nervous system (20.5% vs. 7.9% [12.6% difference]), liver cancer (16.9% vs. 4.7% [12.2% difference]), renal failure (16.4% vs. 4.3% [12.1% difference]), and hypertension (26.1% vs. 18.8% [7.3% difference]). Among AHP patients age ≥ 55 years, the most common long-term complications were hypertension, kidney failure, and liver cancer.

**Conclusions:**

In Japan, patients with AHP experience a high clinical burden in terms of acute attacks, chronic symptoms, and long-term complications. The clinical burden related to chronic symptoms and long-term complications was substantially higher in Japanese patients with AHP compared with a matched population without AHP. Recognizing these signs and symptoms of AHP may aid physicians in making an earlier diagnosis, which may help patients avoid attack triggers, implement disease management, and reduce lifetime disease burden.

## Background

Acute hepatic porphyria (AHP) is a family of rare genetic diseases of heme biosynthesis [1–3]. These diseases, which include autosomal-dominant acute intermittent porphyria (AIP), variegate porphyria (VP), hereditary coproporphyria (HCP), and the autosomal-recessive delta-aminolevulinic acid dehydratase-deficient porphyria, are characterized by acute neurovisceral attacks, chronic symptoms, and long-term complications [1–3]. In Europe, the prevalence of symptomatic AHP is estimated to be 0.5 case per 100,000 population [4]. Recent genetic screening has indicated that clinical penetrance of symptomatic AHP is ~ 1% in the general population [5]. Substantial urinary porphobilinogen elevation (> 3 × upper limit of normal) is a diagnostic feature of AHP, as elevations of this magnitude are not associated with diseases other than AIP, VP, and HCP [3, 6].

Acute AHP attacks can be life-threatening [6]. Severe, incapacitating abdominal pain is typical, and a variety of other symptoms that are suggestive of multisystemic effects may also be present, including gastrointestinal (e.g., nausea, vomiting), autonomic (e.g., tachycardia, hypertension), neurologic/psychiatric (e.g., mental status changes, anxiety), and others (e.g., weakness, hyponatremia) [1, 7–9]. Patients who experience acute attacks often require hospitalization and treatment with opioids to manage abdominal and other types of pain [7]. Aside from acute attacks, patients with AHP experience chronic symptoms and long-term complications that impact quality of life, including prolonged anxiety and depression, hypertension, liver damage, and other organ involvement [8, 10–12]. Altogether, these symptoms have a large impact on patient quality of life and outcomes [13, 14].

Before the approval of givosiran, long-term AHP symptom management was limited to trigger avoidance, suppression of ovulation, and off-label intravenous hemin prophylaxis [7]. Repeated, prophylactic use of hemin may be associated with a number of adverse events, including venous damage, thrombophlebitis, coagulation abnormalities, and secondary iron overload [2, 15]. Givosiran is a subcutaneously administered RNA interference therapeutic that is approved for the treatment of AHP in adults (United States, Brazil, Canada) and adults and adolescents age ≥ 12 years (Japan, European Economic Area, Switzerland) [16–18]. In the phase 3, double-blind ENVISION trial (NCT03338816), givosiran treatment was associated with significant reductions in annualized attack rate, use of hemin for attacks, and levels of the heme intermediates delta-aminolevulinic acid and porphobilinogen, as well as improvements in daily worst pain, compared with placebo [19].

Despite the debilitating symptoms of AHP, few longitudinal studies have been conducted to assess the impact of this disease and long-term complications associated with AHP in these patients, possibly because a diagnosis of AHP is often severely delayed [20]. Notably, an observational study of 108 patients with AHP in the United States found that the mean time from the onset of symptoms to a definitive diagnosis was 15 years [20].

The EXPLORE study, conducted in the United States and Europe, was designed as a prospective, observational study to characterize the current clinical management of patients with AHP who experience recurrent attacks (≥ 3 attacks within preceding 12 months) or are receiving prophylactic treatment [7]. In the cohort of 112 patients studied, 77% (371/483) of acute attacks were severe enough to require urgent care in a health care facility and/or treatment with intravenous hemin [7]. Additionally, approximately two-thirds of patients reported chronic symptoms between attacks, with nearly half reporting daily symptoms, which were experienced regardless of whether the patients were on hemin prophylaxis [7]. Another observational study—this one with a retrospective design—investigated the difference in severity of porphyria attacks before and after diagnosis in a small cohort of Israeli patients (N = 9) and showed that the attacks occurring prior to AHP diagnosis were much more severe than those that occurred after AHP diagnosis [21].

The objective of the present study was to provide an overview of the clinical features of AHP in Japan, including acute attacks, chronic symptoms, and long-term complications, using a nationwide health care claims database. This information may assist physicians in understanding current practice patterns and in driving early detection and diagnosis of AHP in this at-risk population.

## Results

**Fig. 1:**
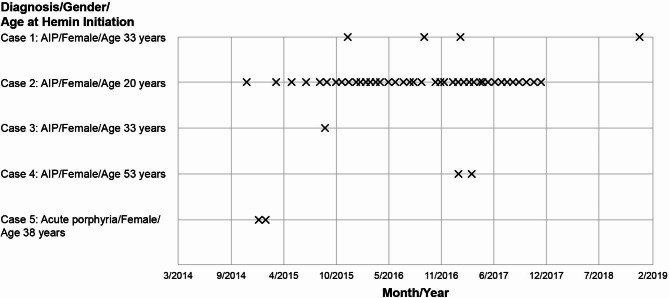
Hemin administration during observation period. AIP, acute intermittent porphyria

A total of 391 patients with AHP were included during the study period (April 2008–June 2020). As Table 1 shows, the mean age at diagnosis was 44.4 years. During the mean observation period of 35.2 months, 28.6% (112/391) of patients experienced acute attacks, with 18.2% (71/391) of patients experiencing 1 attack and 10.5% (41/391) experiencing ≥ 2 attacks. Table 1 also summarizes data regarding patients with acute attacks. Overall, 6.1% of the patients (24/391) had an emergency department (ED) visit and 25.6% (100/391) were hospitalized. There was 1 patient who experienced 11 acute attacks during the observation period. Among patients with multiple attacks, the average time between 2 or more attacks was 281 days. The median time between acute attacks was 183 days among those patients with more than 1 recorded visit (Table 1). Across porphyria types, patients with AIP experienced a higher incidence rate and absolute number of attacks compared with other AHP types. The results of the 1:10 matching for patients with AHP (N = 391) and without AHP (N = 3910) demonstrate comparable mean ages, sex distributions, and observation periods (Table 1). Among all patients, intravenous hemin was administered only to 5 patients, all of whom were female (Fig. 1).

**Table 1:** Patient demographics at time of AHP diagnosis and acute attacks during observation period

		Overall AHP Population	Type			Matched Population
			Acute Intermittent Porphyria	Hereditary Coproporphyria	Variegate Porphyria	
Patients, n		391	120	46	11	3910
Sex, n (%)	Male	177 (45.3)	43 (35.8)	27 (58.7)	6 (54.5)	1770 (45.3)
	Female	214 (54.7)	77 (64.2)	19 (41.3)	5 (45.5)	2140 (54.7)
Age, years	Mean (SD)	44.4 (23.1)	42.6 (19.0)	34.4 (27.1)	56.1 (15.1)	44.8 (23.4)
	Median	44.0	39.5	25.5	57.0	45.0
	Min–max	0–94	11–94	0–88	31–73	0–98
Observation period (first visit~last visit), months	Mean (SD)	35.2 (31.5)	33.0 (31.1)	36.1 (40.9)	41.1 (41.1)	38.3 (34.2)
	Median	27.0	20.5	20.5	34.0	32.0
	Q1–Q3	5.0–59.0	5.0–54.5	3.0–54.0	2.0–72.0	4.0–64.0
	Min–max	1–145	1–135	1–145	1–126	1–147
Patients who had an acute attack that led to an ED visit or admission, n (%)	ED visit	24 (6.1)	15 (12.5)	0 (0.0)	0 (0.0)	—
	ED admission	100 (25.6)	34 (28.3)	11 (23.9)	2 (18.2)	—
Patients who experienced an acute attack, n (%)	Total	112 (28.6)	44 (36.7)	11 (23.9)	2 (18.2)	—
	1	71 (18.2)	23 (19.2)	10 (21.7)	2 (18.2)	—
	≥ 2	41 (10.5)	21 (17.5)	1 (2.2)	0 (0.0)	—
	Cumulative total	207	94	12	2	—
	Mean (SD)	0.5 (1.2)	0.8 (1.6)	0.3 (0.5)	0.2 (0.4)	—
	Min–max	0–11	0–11	0–2	0–1	—
Days to acute attack in patients with ≥ 2 acute attacks	n	95	50	1	0	—
	Mean (SD)	281.3 (339.4)	317.2 (337.7)	21.0 (—)	—	—
	Median	183.0	250.0	21.0	—	—
	Q1–Q3	34.0–439.0	45.0–470.0	21.0–21.0	—	—
	Min–max	1–1810	1–1499	21–21	—	—

AHP, acute hepatic porphyria; ED, emergency department; max, maximum; min, minimum; Q, quintile; SD, standard deviation

Chronic symptoms were also prevalent during the observation period. The most commonly recorded symptoms in the AHP population were sleep disorders (23.0% [90/391]); neurotic, stress-related, and somatoform disorders (21.7% [85/391]); nausea and vomiting, excluding chemotherapy-induced emesis (17.9% [70/391]); other and unspecified abdominal pain (13.6% [53/391]); and mood [affective] disorders (10.7% [42/391]) (Table 2). In the Japanese Medical Data Vision (MDV) database, incidence rates were ~ 10% or higher in the AHP population compared with the matched population for many chronic symptoms, including (from greatest to smallest difference in expression rate) neurotic, stress-related, and somatoform disorders (21.7% vs. 6.7% [15.0% difference in expression rate]); sleep disorders (23.0% vs. 9.9% [13.1% difference]); other and unspecified abdominal pain (13.6% vs. 3.7% [9.9% difference]); and nausea and vomiting, excluding chemotherapy-induced emesis (17.9% vs. 8.1% [9.8% difference]). Some chronic symptoms, including nausea, may also manifest during acute attacks.

**Table 2:** Incidence of chronic symptoms during observation period

	Overall AHP Population, N (%)	Matched Population, N (%)
**Chronic symptoms (** ***ICD-10*** **)**	391	3910
**Abdominal pain**		
Pain localized to upper abdomen (R10.1)	17 (4.3)	49 (1.3)
Pelvic and perineal pain (R10.2)	1 (0.3)	8 (0.2)
Pain localized to other parts of lower abdomen (R10.3)	9 (2.3)	62 (1.6)
Other and unspecified abdominal pain (R10.4)	53 (13.6)	143 (3.7)
**Muscle pain**		
Myalgia (M79.1)	13 (3.3)	73 (1.9)
**Low muscle strength**		
Other symptoms and signs involving nervous and musculoskeletal systems (R29.8)	9 (2.3)	12 (0.3)
**Nausea**		
Nausea and vomiting (excluding chemotherapy-induced emesis) (R11 except R11 Z512)	70 (17.9)	318 (8.1)
**Fatigue**		
Malaise and fatigue (R53)	7 (1.8)	27 (0.7)
**Insomnia**		
Sleep disorders (G47.0)	90 (23.0)	389 (9.9)
**Mental disorder**		
Mood [affective] disorders (F30-F39)	42 (10.7)	130 (3.3)
Neurotic, stress-related, and somatoform disorders (F40-F48)	85 (21.7)	261 (6.7)
Behavioral syndromes associated with physiological disturbances and physical factors (F50-F59)	12 (3.1)	24 (0.6)
**Mental disorder (details)**		
Manic episode (F30)	0 (0)	0 (0)
Bipolar affective disorder (F31)	6 (1.5)	14 (0.4)
Depressive episode (F32)	35 (9.0)	112 (2.9)
Recurrent depressive disorder (F33)	0 (0)	2 (0.1)
Persistent mood [affective] disorders (F34)	2 (0.5)	12 (0.3)
Other mood [affective] disorders (F38)	0 (0)	1 (0)
Unspecified mood [affective] disorder (F39)	0 (0)	0 (0)
Phobic anxiety disorders (F40)	0 (0)	1 (0)
Other anxiety disorders (F41)	44 (11.3)	133 (3.4)
Obsessive-compulsive disorder (F42)	2 (0.5)	3 (0.1)
Reaction to severe stress, and adjustment disorders (F43)	6 (1.5)	13 (0.3)
Dissociative [conversion] disorders (F44)	15 (3.8)	13 (0.3)
Somatoform disorders (F45)	29 (7.4)	85 (2.2)
Other neurotic disorders (F48)	26 (6.6)	60 (1.5)
Eating disorders (F50)	10 (2.6)	11 (0.3)
Nonorganic sleep disorders (F51)	1 (0.3)	8 (0.2)
Sexual dysfunction, not caused by organic disorder or disease (F52)	0 (0)	2 (0.1)
Mental and behavioral disorders associated with puerperium, not elsewhere classified (F53)	0 (0)	0 (0)
Psychological and behavioral factors associated with disorders or diseases classified elsewhere (F54)	1 (0.3)	3 (0.1)
Abuse of non-dependence-producing substances (F55)	0 (0)	0 (0)
Unspecified behavioral syndromes associated with physiological disturbances and physical factors (F59)	0 (0)	0 (0)

AHP, acute hepatic porphyria; *ICD-10, International Classification of Diseases, Tenth Revision;* MDV, Medical Data Vision

Long-term complications were also prevalent among patients with AHP. The incidence of long-term complications during the observation period was 26.1% (102/391) for hypertension; 20.5% (80/391) for polyneuropathies and other disorders of the peripheral nervous system; 16.9% (66/391) for malignant neoplasms of the liver and intrahepatic bile ducts, and hepatocellular carcinoma; 16.4% (64/391) for renal failure; and 15.9% (62/391) for hepatic fibrosis and cirrhosis (Table 3). Higher incidence rates for long-term complications were seen in the AHP population compared with the matched population; these complications (listed from greatest to smallest difference in incidence) included fibrosis and cirrhosis of liver (15.9% vs. 3.0% [12.9% difference in expression rate]), polyneuropathies and other disorders of the peripheral nervous system (20.5% vs. 7.9% [12.6% difference]), liver cancer (16.9% vs. 4.7% [12.2% difference]), renal failure (16.4% vs. 4.3% [12.1% difference]), and hypertension (26.1% vs. 18.8% [7.3% difference]) (Table 3). Among patients with long-term complications that started or were persistent at age ≥ 55 years, the observed incidences of liver cancer, kidney failure, chronic kidney disease, and hypertension were ≥ 10% greater than in the overall AHP population (Table 3).

**Table 3:** Incidence of long-term complications during observation period

	Overall AHP Population				AHP Population Age ≥ 55 Years				Matched Population
	Overall	Type			Overall	Type			
		AIP	HCP	VP		AIP	HCP	VP	
Patients, n	391	120	46	11	149	34	15	8	3910
Complications for observation period (*ICD-10*), n (%)									
*Hepatitis, cirrhosis of liver, liver cell carcinoma*									
Hepatic failure, not elsewhere classified (K72)	26 (6.6)	7 (5.8)	2 (4.3)	1 (9.1)	15 (10.1)	2 (5.9)	1 (6.7)	1 (12.5)	31 (0.8)
Chronic hepatitis, not elsewhere classified (K73)	28 (7.2)	3 (2.5)	3 (6.5)	2 (18.2)	11 (7.4)	0 (0.0)	1 (6.7)	2 (25.0)	51 (1.3)
Fibrosis and cirrhosis of liver (K74)	62 (15.9)	13 (10.8)	6 (13.0)	1 (9.1)	31 (20.8)	5 (14.7)	3 (20.0)	1 (12.5)	118 (3.0)
Malignant neoplasm of liver and intrahepatic bile ducts, liver cell carcinoma (C22.0)	66 (16.9)	9 (7.5)	12 (26.1)	2 (18.2)	43 (28.9)	5 (14.7)	8 (53.3)	2 (25.0)	183 (4.7)
*Kidney disease*									
Kidney failure (N17-N19)	64 (16.4)	19 (15.8)	4 (8.7)	0 (0.0)	47 (31.5)	11 (32.4)	4 (26.7)	0 (0.0)	167 (4.3)
Acute kidney failure (N17)	10 (2.6)	4 (3.3)	2 (4.3)	0 (0.0)	7 (4.7)	1 (2.9)	2 (13.3)	0 (0.0)	40 (1.0)
Chronic kidney disease (N18)	33 (8.4)	6 (5.0)	2 (4.3)	0 (0.0)	28 (18.8)	5 (14.7)	2 (13.3)	0 (0.0)	100 (2.6)
Unspecified kidney failure (N19)	31 (7.9)	13 (10.8)	1 (2.2)	0 (0.0)	20 (13.4)	7 (20.6)	1 (6.7)	0 (0.0)	79 (2.0)
*Nerve injury*									
Polyneuropathies and other disorders of peripheral nervous system (G60-G64)	80 (20.5)	33 (27.5)	7 (15.2)	2 (18.2)	38 (25.5)	15 (44.1)	5 (33.3)	1 (12.5)	308 (7.9)
Hereditary and idiopathic neuropathy (G60)	0 (0.0)	0 (0.0)	0 (0.0)	0 (0.0)	0 (0.0)	0 (0.0)	0 (0.0)	0 (0.0)	0 (0.0)
Inflammatory polyneuropathy (G61)	25 (6.4)	11 (9.2)	2 (4.3)	1 (9.1)	7 (4.7)	3 (8.8)	1 (6.7)	0 (0.0)	3 (0.1)
Other polyneuropathies (G62)	63 (16.1)	26 (21.7)	6 (13.0)	2 (18.2)	28 (18.8)	11 (32.4)	4 (26.7)	1 (12.5)	238 (6.1)
Other disorders of peripheral nervous system (G64)	12 (3.1)	1 (0.8)	3 (6.5)	0 (0.0)	6 (4.0)	1 (2.9)	2 (13.3)	0 (0.0)	98 (2.5)
*Hypertension*									
Essential (primary) hypertension (I10)	102 (26.1)	27 (22.5)	9 (19.6)	4 (36.4)	77 (51.7)	18 (52.9)	8 (53.3)	4 (50.0)	736 (18.8)

AHP, acute hepatic porphyria; AIP, acute intermittent porphyria; AP, acute porphyria; HCP, hereditary coproporphyria; HP, hepatic porphyria; *ICD-10, International Classification of Diseases, Tenth Revision;* MDV, Medical Data Vision; VP, variegate porphyria

## Discussion

This research highlights the high clinical impact facing patients with AHP in Japan. This impact is characterized by acute attacks, chronic symptoms, and long-term complications. While AHP is rare, the most common presenting symptoms are nonspecific and acute. Chronic symptoms such as somatoform and sleep disorders, and long-term complications such as liver fibrosis, cirrhosis, and liver cancer, also occur at a higher frequency in this population versus the general population.

This population of patients with AHP in Japan may have milder disease than a previously studied population of patients with AHP. For example, Gouya et al. [7] reported that 88% of the patients in their study experienced acute attacks, 77% of whom required treatment at a health care facility and/or hemin administration, compared with 29% of patients who experienced acute attacks in the present study. This disparity may also be attributable to a difference in defining acute attacks; in the natural history study by Gouya et al., acute attacks that occurred at home were recorded, whereas in the present study, only acute attacks that required an ED visit or admission were recorded in the database. Rates of chronic symptoms reported in prior studies were also higher than those observed in the present study. Gouya et al. [7] reported that chronic symptoms between attacks were seen in 65% (73/112) of patients with AHP who had 3 or more attacks per year or who were receiving prophylactic treatment; the most common of these chronic symptoms were pain, nausea, and fatigue. In a chart review of Spanish patients with AIP, Buendía-Martínez et al. [22] reported that chronic symptoms were seen in 85% (23/27) of patients with fewer than 4 acute attacks per year and in 46% (13/28) of patients without previous neurogenic attacks. The most common symptoms in their study were nonspecific abdominal pain, fatigue, myalgia, anxiety, and insomnia. The present study was conducted using data from a national health care claims database; given the previous study results, the proportion of patients actually experiencing chronic symptoms is probably higher than the rate observed in the present study.

The incidence rates of long-term complications found in the present study were lower than those reported by Buendía-Martinez et al. [22]. The incidence rates of both chronic kidney disease and hypertension were higher in the AIP population studied previously, compared with the Japanese population with AIP in the present study (29.6% vs. 5.0% and 33.3% vs. 19.6%, respectively). The present study also compared complication rates between age groups and found that, within the AHP population, certain complications, such as kidney failure and liver cancer, had a higher incidence among patients age ≥ 55 years. While the incidence of long-term complications such as cancer is expected to increase with age (even in a non-AHP population) because of the heme biosynthesis pathway dysfunction that defines AHP and the resulting buildup of harmful porphyrins, it is possible that the higher incidence of complications in the older subgroup may be in part attributable to AHP. This may be especially true for complications (e.g., liver cancer) that include organ involvement directly related to the dysfunction of the heme biosynthesis pathway [23]. This suggests that the onset of symptoms may occur long before patients receive a confirmed diagnosis, highlighting the importance of early detection and management.

This study has several strengths. Data on non-attack symptoms and long-term complications in patients with non-severe AHP is limited in the literature, and this study adds clinical evidence from a less severe subset of the AHP population that also exhibited substantial disease burden and clinical impact. Additionally, many studies on the epidemiology of AHP focus on patients with AIP; however, the present study adds evidence regarding acute attacks and long-term complications in patients with multiple types of AHP, which permits us to compare outcomes across diagnoses. For example, among patients with AIP, the incidence of acute attacks was ~ 20% higher than the rate among patients with HCP and VP. Similarly, this study provides clinical evidence from a single cohort of Japanese patients, which is important for understanding the impact of AHP on patients worldwide with varying demographics.

This study also has limitations. First, the MDV database does not include data from every hospital in Japan; it is possible that some patients received diagnoses and treatments at hospitals that do not contribute data to the MDV database [24]. Second, the MDV database does not capture all laboratory data, and genetic testing was not covered by national insurance until April 2022. Therefore, AHP diagnosis cannot be confirmed directly in all cases based on the information available in the database records [24]. However, Japanese diagnostic criteria preclude AHP diagnosis based on clinical characteristics alone; confirmatory results of laboratory and/or genetic tests, and exclusion of other diagnoses, are also required [25]. Third, AHP could have been diagnosed at any time during the study period. Hence, in some cases, some of the outcomes studied may have been unrelated to AHP (e.g., outcomes that occurred within the study period, but before AHP diagnosis). However, given that there is an on-average delay of about 15 years between AHP symptom onset and diagnosis [20], it is likely that much of the observed health care resource utilization and clinical outcomes were related to AHP. Fourth, this study was descriptive in nature; patients with AHP were matched to patients without AHP in the general population, and statistical testing was not performed. Finally, there are limitations associated with the use of administrative data, including the possibility of coding errors and misclassification. For example, the presence of a diagnosis code in a medical record may be related to an attempt to rule out a given condition, rather than a diagnosis or treatment for that condition. Additionally, it is possible that the population represented in the MDV database is not representative of the broader Japanese population, as not all hospitals contribute data to the database. However, for AHP and other rare diseases, which have clinical research studies limited by missing data and small sample sizes, administrative data are especially useful. Administrative data analyses add to the AHP literature and point to additional areas for future research to better define the long-term trajectory of disease in patients with AHP, and potential benefits of earlier diagnosis and proactive management of AHP.

## Conclusions

In Japan, patients with AHP experience a high clinical burden in terms of acute attacks, chronic symptoms, and long-term complications. An early diagnosis is critical to avoiding acute attack triggers, implementing a disease management plan, and reducing lifetime disease burden. Recognition of these non-specific symptoms and complications may aid physicians and other health care providers in suspecting and evaluating patients for AHP in daily practice and achieving early diagnosis of this disease.

## Methods

### Data source

We aimed to provide an overview of the clinical features of AHP in Japan in order to assist physicians in understanding current practice patterns and to facilitate early detection and diagnosis of AHP in this at-risk population. This retrospective cohort study used the MDV health care claims database. As of August 2020, data from 32.6 million patients had been collected from 419 acute care hospitals across Japan, with 10 million hospital visits recorded in the prior year. These data represent ~ 9% of the total Japanese population and ~ 24% of all acute care hospitals in Japan.

## Patient selection

Patients in the MDV database with porphyria claims data were selected using *International Classification of Diseases, Tenth Revision* (*ICD-10*) codes between April 2008 and June 2020 (N = 1430). Patients with hereditary erythropoietic porphyria, erythropoietic protoporphyria, protoporphyria, porphyria cutanea tarda, porphyria, and congenital porphyria were excluded to extract patients with AHP. Each patient’s diagnosis status was marked in the MDV database with a flag indicating “suspected” or “confirmed,” which was determined by a physician. The Japan Intractable Diseases Information Center developed diagnostic criteria for AHP based on the results of clinical, laboratory, and genetic tests [24, 25]. As genetic testing was not covered by national insurance until April 2022, and laboratory data were collected from a limited number of institutions, the MDV database does not include genetic testing data [24, 26]. As a result, in the present study, AHP cases included both confirmed and suspected cases. For patients with multiple porphyria diagnoses, the latest diagnosis was included in the overall patient sample count.

## Study definitions

Time of diagnosis, first and final visit, and observation period were recorded for each patient. The time of diagnosis was defined as the month and year when AHP was diagnosed. If a suspected diagnosis was made and then later confirmed, the time of diagnosis was defined as the month and year during which the diagnosis was first made. If a patient had multiple confirmed diagnoses, the time of diagnosis was defined as the month and year of the first confirmed diagnosis. If a patient had multiple suspected diagnoses, the time of diagnosis was defined as the month and year of the most recent suspected diagnosis. The first visit was defined as the first visit to a health care facility documented during the entire study period, and the final visit was defined as the final facility visit during the study period. The observation period was defined as the time between the first and final visits.

## Outcomes assessed

Outcomes were identified using *ICD-10* codes, and were evaluated over the entire study period regardless of the time of AHP diagnosis. The outcomes of interest included acute attacks, chronic symptoms, and long-term complications. Acute attacks were defined as ED visits or admissions. Chronic symptoms were defined and categorized as abdominal pain, muscle pain, muscle weakness, nausea, fatigue, anxiety, insomnia, and mental health symptoms [22]. Long-term complications were documented among patients with AHP during the observation period and compared with those of a matched population—each patient with AHP was matched with 10 patients without AHP of the same sex and age group—in the same year as the month of the visit when the patient’s AHP age was obtained. Long-term complications were categorized as liver disease, chronic kidney disease, hypertension, and nervous system disorders. Complications were evaluated separately among patients with a diagnosis of AIP, HCP, or VP. Additionally, complications among patients age ≥ 55 years were also evaluated separately to assess the difference in complication rates between premenopausal and postmenopausal women.

## Data Availability

Not applicable; database study using deidentified and anonymous data.
